# The meanings of cultural competence in mental health: an exploratory focus group study with patients, clinicians, and administrators

**DOI:** 10.1186/s40064-016-2037-4

**Published:** 2016-03-31

**Authors:** Neil Krishan Aggarwal, Kryst Cedeño, Peter Guarnaccia, Arthur Kleinman, Roberto Lewis-Fernández

**Affiliations:** Columbia University, 1051 Riverside Drive, Unit 11, New York, NY 10032 USA; New York State Psychiatric Institute, New York, NY USA; Department of Human Ecology, Rutgers, The State University of New Jersey, Newark, NJ USA; Department of Social Medicine, Harvard University, Cambridge, MA USA; Columbia University Medical Center, New York, NY USA; NYS Center of Excellence for Cultural Competence, and Hispanic Treatment Program, New York State Psychiatric Institute, New York, NY USA

**Keywords:** Cultural competence, Cultural sensitivity, Cultural psychiatry, Cross-cultural psychiatry, Transcultural psychiatry, Focus groups, Document analysis, Policy analysis

## Abstract

Cultural competence training is mandatory in the United States of America to alleviate minority health disparities though few studies have examined perceptions across stakeholders. We conducted separate focus groups with patients, clinicians, and administrators from the psychiatry department at one community hospital and compared responses to hospital policies. Stakeholders defined cultural competence through group-based or person-centered traits despite policies recommended person-centered approaches. Administrators and clinicians named clinician techniques for psycho-education whereas patients named these techniques for enlistment in treatment planning as equals. All groups named patient cultural views and institutional challenges as barriers to care, but only patients and administrators additionally named clinician biases as possible barriers. We discuss these discrepant perceptions and possible solutions to improve research, practice, and policy on cultural competence in mental health.

## Background

The Office of the U.S. Surgeon General (2001) and the Institute of Medicine (2003) have contended that cultural competence training for clinicians can help close disparities for racial and ethnic minorities. Cultural competence training is required during medical school (Association of American Medical Colleges 2015) and residency training in all specialties (Ambrose et al. 2013). Professional societies for psychiatrists (American Psychiatric Association 2013), psychologists (American Psychological Association 2002), and social workers (National Association of Social Workers 2001) also recommend cultural competence training for independent practitioners. The sociologist Durkheim (2013) considered social facts to be types of knowledge and behavior with coercive power over individuals, and cultural competence training has now become a social fact to cultivate ethical behavior (Shaw and Armin 2011) through professional and legal mandates.

Social scientists have since identified numerous understandings of culture in cultural competence trainings. In the absence of a consensus definition for culture in the medical field, administrators have implemented cultural competence in various ways, from low-cost celebrations of patient festivals to the high-cost hiring of translators (Guarnaccia and Rodriguez 1996). Many trainings treat culture as a negative set of group traits that prevents minority patients from adhering to treatments recommended by clinicians rather than as a dynamic process through which all people make meanings of health and illness (Carpenter-Song et al. 2007; Kleinman and Benson 2006). Clinicians may view these trainings as required forms of political correctness that can be practically removed from patient care (Bullon 2013; Willen 2013), reinforced by the use of exotic cases that may stereotype patients (Jenks 2011). Based on these findings, a Lancet Commission (Napier et al. 2014) and the National Institute of Health (Kagawa-Singer et al. 2014) have recommended research on improving cultural competence trainings based on stakeholder participation.

This article examines how patients, clinicians, and administrators define the meanings and practices of cultural competence in one hospital. No published study has examined patient perspectives on clinician cultural competence in mental health or compared perspectives across stakeholders. We engaged participants at one hospital to investigate how knowledge and practice is locally constructed and contested, in line with the exploratory focus of qualitative health research (Lambert and McKevitt 2012). This article’s aims are to: (1) compare themes within and across three focus groups conducted separately with patients, clinicians, and administrators, and (2) assess how these themes relate to cultural competence policies at the hospital study site.

## Methods

### Design

We stratified participants through theoretical sampling into separate focus groups for patients, clinicians, and administrators. Theoretical sampling achieves data saturation quicker than convenience or random sampling based on the assumption of a group’s coherence (Morse 1995). Two forms of data justified theoretical sampling by stakeholder in this study. First, these three groups are differentially impacted by health interventions (Damschroder et al. 2009; Llerena-Quinn 2013). Second, the first author met with clinicians and administrators before initiating the study, and they asked for separate groups to speak freely without repercussions. Compared to individual interviews or expert consensus panels (Kitzinger 1995; Morgan 1996), focus groups uniquely allowed us to observe how participants query and explain cultural competence to each other.

### Sample

All participants were recruited from the psychiatry department of a major metropolitan hospital in Queens, NY, USA. We selected this hospital because it provides care in a setting of *hyperdiversity*. Hyperdiversity is a complex environment characterized by social differences beyond just race and ethnicity that includes national origin, immigration status, and linguistic group (Good and Hannah 2015). According to U.S. Census (2015) statistics, no racial or ethnic group has a majority in Queens: Whites are 49.7 %, Asians are 25.2 %, and Blacks are 20.9 % of the population, with Hispanics/Latinos cutting across multiple races at 28 %.

The first author recruited clinicians and administrators through (1) a grand-rounds presentation on cultural competence in mental health, (2) presentations at clinician and administrator staff meetings, and (3) flyers in staff mailboxes. We selected these recruitment strategies based on the recommendations of the study’s main Institutional Review Board (IRB) that forbids authors from directly approaching subjects to avoid coercion in study participation. Therefore, our sampling strategies may have been biased to include only those clinicians and administrators who attend grand rounds and staff meetings in which attendance is not mandatory.

Any clinician who worked part-time or more as identified by the hospital clinical director was eligible for inclusion. Clinicians were defined as psychiatrists, psychologists, social workers, nurses, and credentialed alcoholism and substance abuse counselors (CASACs). Clinicians supervising other clinicians were ineligible for the clinician focus group, but were enrolled in the administrator group. Administrators were defined as any staff members who supervised clinicians and had no exclusion criteria. Fifteen clinicians and administrators of a total possible pool of 30 participated in the focus groups. As hospital employees, clinicians and administrators may have had preexisting relationships that determined who participated in the study, potentially introducing a bias into the study.

Patients were recruited through three types of clinician referrals: (1) the intake coordinator who registered patients in the waiting room could introduce the study before patients met with clinicians, (2) clinicians participating in the study could recruit their patients, and (3) clinicians not participating in the study could also recruit patients. For inclusion, patients had to receive mental health services at the hospital, provide written informed consent after an explanation of study procedures, and self-identify as a racial or ethnic minority. The exclusion criteria for patients were active suicidality, homicidality, intoxication, or a cognition-impairing condition such as dementia, mental retardation, or florid psychosis. As with clinician enrollment, our recruitment strategy may have introduced biases if participating clinicians enrolled patients whom they believed would support clinician and hospital policies on cultural competence, especially if the numbers of patients recruited by participating clinicians exceeded patients recruited by non-participating clinicians. To prevent potential repercussions from hospital administrators who could become upset that clinicians were not recruiting patients, we assured the hospital’s IRB that we would not keep track of which intake coordinators or clinicians recruited patients.

The study protocol received approvals from the IRBs at the New York State Psychiatric Institute (author institution) and Queens Metropolitan Hospital (a pseudonym: henceforth, QMH). All participants provided written informed consent and received $40 in compensation. To prevent any conflict of interest, the researchers are not clinicians at QMH. This professional distance allowed the researchers to ask QMH participants about their experiences with cultural competence, but also prevented them from observing direct patient interactions since they were not credentialed as hospital employees.

### Data collection

The first author coordinated logistics with the chief administrator and clinical director of QMH’s psychiatry department in a preliminary meeting before the focus groups were conducted. Concerns were raised that patients, clinicians, and administrators may not be willing to meet to discuss their experiences without incentives. We agreed to an arrangement in which the first author would offer cash to all participants as an expression of appreciation and QMH would provide sandwiches and soda to incentivize participation during a 90-min lunch period when no clinical services would be offered. All focus groups were conducted in a conference room with chairs in a semi-circle for maximal group interaction.

The first author created focus group guides by identifying themes from systematic literature reviews on cultural competence in mental health (Huey et al. 2014; Renzaho et al. 2013). The guides followed a tunneled approach with introductory questions for all to answer, followed by specific questions based on individual experiences (Morgan and Krueger 1998). Eight to twelve questions were intended for completion in a 90–120 min period (Krueger 1998). Questions were written similarly for all groups in order to permit thematic comparison by stakeholder (Morgan 1996). The last three authors—a medical anthropologist, a psychiatrist-anthropologist, and a cultural psychiatrist, respectively—reviewed the guides for clarity and face validity. All authors have conducted research on cultural competence in mental health settings and have extensive experience in qualitative methods. Table 1 presents questions from the focus group guides.

**Table 1:** Discussion questions from the focus group guide

Discussion topic	Patient questions	Clinician questions	Administrator questions
Welcome and introduction (~10 min)			
General questions (~30 min)			
Defining cultural competence	To break the ice, let’s go around the room and have each person answer. Please tell us what types of services you receive as patients. Please also tell us what cultural competence means to you	To break the ice, let’s go around the room and have each person answer. Please tell us if you are a psychiatrist, psychologist, nurse, social worker, or CASAC. Please also tell us what cultural competence means to you	To break the ice, let’s go around the room and have each person answer. Please tell us what types of services you manage as administrators. Please also tell us what cultural competence means to you
Cultural competence between patients and clinicians	How can a clinician demonstrate cultural competence to patients?	[Same]	[Same]
Beneficiaries of cultural competence	How does a patient’s background affect communication with clinicians? How does a patient’s social, cultural, or racial background affect rapport with clinicians?	How does a patient’s background affect your communication? How does a patient’s social, cultural, or racial background affect your rapport?	[Same as patient question]
Specific questions (30 min)			
Identifying the target population	We want to hear about your specific experiences as patients. Based on your experiences receiving care, for what types of patients is cultural competence necessary and why?	We want to hear about your specific experiences in providing treatment. Based on your experiences with providing care, for what types of patients is cultural competence necessary and why?	We want to hear about your specific experiences as administrators. Based on your experiences managing clinicians, for what types of patients is cultural competence necessary and why?
Cultural competence and patient treatment expectations	Does culture affect what types of treatment you want or how long you want to be treated? If so, how?	Does culture affect what types of treatment patients want or how long they want to be treated? If so, how?	[Same as clinician question]
Cultural competence and clinician healthcare practices	How does cultural competence translate into quality caregiving?	[Same]	[Same]
Resolving patient-clinician differences	If patients and clinicians disagree about what types of treatment are necessary, how should clinicians resolve differences?	[Same]	[Same]
Reflecting back (~10 min)	If you had a chance to give advice on cultural competence to your clinicians, what advice would you give?	If you had a chance to give advice on cultural competence to your clinical director, what advice would you give?	[Same as patient question]
Wrap up and final comments (~10 min)	Is there anything that we should have talked about, but missed in today’s discussion?	[Same]	[Same]

The first author moderated all groups, reading a standardized introduction aloud to explain the study’s purpose, risks, and benefits. The introduction stated that all groups would be taped and transcribed verbatim for data analysis. Participants could opt out at any time; none opted out. All participants completed informed consent and demographic forms. Two tape recorders were placed equidistantly from the ends of the table. Each focus group lasted about 90 min and was in English. Participants were encouraged to speak with the moderator afterward to share content privately; this individual data is not presented here.

### Analytical strategy

All recordings were sent for professional transcription. The second author checked transcripts for accuracy by listening to both recordings per group separately and cleaning errors. The first author reviewed the second author’s transcripts against the original recording to finalize one transcript per group.

We used frequencies and percentages to describe our sample through demographic forms. We used iterative open coding, constant comparison, and neutral questioning for theory generation (Strauss and Corbin 1998). The first two authors independently open-coded one transcript (i.e., patients) to generate coding categories grounded in data. We discussed codes and themes, challenged each other’s interpretations, created a joint codebook, and wrote analytical memos describing new coding categories and subcategories. We used the joint codebook to independently re-code the transcript until achieving an inter-rater reliability >80 % for 10 % of randomly selected codes (Morrison-Beedy et al. 2001). Inter-rater differences were resolved by consensus. Three rounds of independent coding were required to meet this reliability standard for the patient transcript; two rounds were required for the other transcripts. We conducted peer-debriefing sessions, consulted with qualitative experts (our co-authors), and developed an audit trail for trustworthiness and rigor (Strauss and Corbin 1998). We jointly coded conversational process in the transcripts since the benefit of using focus groups as a qualitative method is that they can reveal the process of knowledge construction through participant interactions (Kitzinger 1994; Reed and Payton 1997). Finally, we compared codes across all focus groups to analyze thematic convergences and divergences. We generated queries and reports in NVivo on codes to identify data patterns.

## Results

### Sample characteristics

 Table 2 presents participant characteristics. All groups were comparable across age and duration of residence in the United States. Administrators were trained as master’s level social workers (n = 4), business associate’s degree (n = 2), a nursing manager with an MBA (n = 1), a social worker with a doctorate degree (n = 1), and a CASAC (n = 1). Clinicians were trained as social workers (n = 5), CASACs (n = 3), and a psychiatrist (n = 1). All administrators and clinicians had secondary education compared to about half of the patient sample. Most administrators and clinicians were European-Americans compared to patients, among whom no race predominated. Most administrators (n = 8) and clinicians (n = 7) were female compared to patients (n = 4, 50 %). Almost all administrators spoke only English as a primary language compared to most clinicians and patients who spoke a second language. Most administrators were born in the US compared to most patients born abroad; clinicians were born either in the US or in Asia. Most participants generally practiced Judaism or Christianity.

**Table 2:** Sample characteristics

Characteristics	Administrators (*n* = 9)		Clinicians (*n* = 9)		Patients (*n* = 8)	
	N	Mean (SD)/%	Mean (SD)/%		Mean (SD)/%	
Age	9	52.7 (10.9)	9	48.7 (9.8)	8	50.0 (9.2)
Years of formal schooling						
Beyond high school	9	100.0	9	100.0	4	50.0
<12 years	0	0.0	0	0.0	4	50.0
Gender						
Female	8	88.9	7	77.8	4	50.0
Male	1	11.1	2	22.2	4	50.0
Race/ethnicity						
Caucasian	5	55.5	5	55.5	1	12.5
Hispanic/Latino/a	2	22.2	0	0.0	2	25.0
Black/African American	1	11.1	1	11.1	1	12.5
Asian	1	11.1	3	33.3	2	25.0
Other^a^	0	0.0	0	0.0	1	12.5
Primary language						
English	8	88.9	5	55.5	6	75.0
Malayalam	1	11.1	0	0.0	0	0.0
Telugu	0	0.0	1	11.1	0	0.0
Bengali	0	0.0	1	11.1	0	0.0
Korean	0	0.0	1	11.1	0	0.0
Russian	0	0.0	1	11.1	0	0.0
Spanish	0	0.0	0	0.0	1	12.5
Mandarin	0	0.0	0	0.0	1	12.5
Secondary language						
None	7	77.8	4	44.4	3	37.5
English	1	11.1	4	44.4	2	25.0
Other	1	11.1	1	11.1	3	37.5
Foreign born	3	33.3	4	44.4	4	50.0
Years of residence in US^b^	8	44.5 (17.0)	8	41.1 (16.3)	8	44.4 (14.2)
Religion^c^						
Jewish	3	33.3	1	11.1	0	0.0
Christian	1	11.1	1	11.1	0	0.0
Other	5	55.5	7	77.8	7	87.5
None	0	0.0	0	0.0	1	12.5

^a^“Other” includes bi- and multi-racial participants

^b^One administrator, and one clinician did not answer

^c^One patient did not answer

### Themes

Table 3 summarizes themes and subthemes identified through coding. Participants discussed five themes around culturally competent care (CCC): (1) definitions, (2) clinician techniques, (3) patient challenges, (4) clinician challenges, and (5) institutional challenges.

**Table 3:** Themes regarding culturally competent care

Theme	Code	Representative quote	Focus group
Definitions	Demographic	The appreciation of people where they come from, their heritage, faith, and belief systems	P, C, A
	Person-centered	Relating to the patient based on their background, and taking into consideration where they’re from	P, C, A
Clinician techniques	Sharing similarities	Instead of the differences I would focus more on the things that we have in common	P, C, A
	Respect patient wishes	Well, that’s up to the patient to make that decision	P, A
	Explain options to patients	If it doesn’t make it better for you, come back and tell me and we’ll find something different	P, C, A
Patient challenges	Cultural model of mental health	I think that our country is very Spanish, so if you go to the *psiquiatra* (psychiatrist) or the *psicólogo* (psychologist) it means you’re crazy	P, C
	Cultural view of mental illness	I can’t think of any culture where mental illness isn’t a stigma	P, C
	Concerns about the clinician’s culture	Some patients reject you for who we are because we are from another culture	C, A
Clinician challenges	Explicit bias	I had one therapist that, I’m mixed, and when I would come to see her, all she wanted to talk about was my culture	P, A
	Implicit bias	We all have attitudes and beliefs that we come into this practice with that we have to check at the door and be very mindful of	P, A
Institutional challenges	Time	A long time ago you would say 45 minutes, but now it’s half an hour and hopefully they don’t cut that	P, C, A
	Technological pressures	Before technology, the therapist would look at you. But now they’re typing while you’re talking to them. You don’t feel it	P, C

*P* patients, *C* clinicians, *A* administrators

#### CCC definitions

All participants defined culturally competent care as (1) acknowledging group-based demographic traits of patients, or (2) delivering person-centered care based on individual characteristics, though clear differences emerged by stakeholder. Seven of nine administrators focused on group demographic traits whereas six of nine clinicians focused on person-centered care. Seven of eight patients focused on person-centered care, but in ways that differed from clinicians. One patient simply stated, “I don’t know.”

Definitions based on group demographic traits began with attempts to identify markers of identity as clinically relevant. One administrator typified the list of traits elicited: “Cultural competence would be understanding different beliefs, different religions, different languages, and understanding that we have different cultures.” The word *background* appeared frequently among administrators to describe culture as a set of inherited traits: “Cultural competence is the ability to work with patients from all different cultural backgrounds, religious backgrounds, and the ability to also lean on and rely on our colleagues who are from different cultures in helping us deal with certain patients. If they’re not from our own cultural background, we can rely on others for help as well.”

Some administrators and clinicians discussed ways to treat group traits as targets for clinical intervention. One administrator explained the medicalization of cultural knowledge in the hospital: “Cultural competence means the ability to understand [the] different cultures of people. In nursing, we have meetings on different cultures. Staff talk about, ‘That’s what you do with Chinese. With Koreans you do this.’ Things like that so that we can learn. In our orientation time, we have a little talk about culture.” Another administrator explained interventions by group: “Hispanic people have kind of personality types or needs that needs to be met. For example, they want to feel like when they come to see anybody, it feels like family. It feels like they can trust.” Three clinicians also defined cultural competence through group traits related to clinical practice. For one clinician, traits informed communication: “I had this patient who was from the Asian culture. He had a lot of problem with eye contact. What I would do is that I would not make that much eye contact with that particular patient until he became more comfortable.” For another clinician, traits determined family involvement: “Most of the Asian cultures have nuclear families. They want the family to be included. Mothers and fathers, you need to speak to them first.” For the third clinician, traits influenced treatment: “In the African-American community, it’s a shame to have a mental illness. The best kind of treatment for an African American is called brief therapy. Make it quick, give them their medication. They don’t want to try and go all into the dynamics of the disease or the biopsychosocial aspects of it.”

In contrast, definitions based on person-centered care expressed the need to overcome differences in the identities of all individuals. One patient with schizophrenia represented other patient responses: “It’s respecting everybody’s culture. Just because you don’t speak the same language—It’s not that you don’t have a brain to understand, it’s that you don’t understand that language. I continue to take medication and see any doctor—Asian doctor, Spanish doctor, I don’t mind—who speaks English.” Clinicians answered similarly, typified by this psychiatrist: “Cultural competence means understanding what their belief system is like and to be sensitive to their needs and also how they present their symptoms to us. Everybody is different in the way they perceive their symptoms and it important for us to understand so we can be able to approach them properly.” Only two administrators defined cultural competence in this manner.

Two patients and a single clinician—but no administrators—defined culturally competent care as fighting stigma. One patient with bipolar depression exemplified these responses of cultural competence as “more education on mental illness so that people can understand that because you have a mental illness, that doesn’t mean that you are a crazy person, that you have to be hospitalized for life.” A patient with a different disorder said that cultural competence is “enlightening people on mental issues so that people don’t stigmatize.”

#### Clinician techniques for CCC

Patients, clinicians, and administrators identified clinician techniques for culturally competent care. All groups mentioned (1) sharing similarities and (2) explaining options to patients, but only patients and clinicians named (3) respecting patient wishes.

Patients wanted clinicians to reveal personal information in sharing similarities. One patient said that clinicians should be “relating with their own cultures and associating what may seem the same, like beliefs or upbringings or child rearing, so that people feel more comfortable.” Three clinicians agreed, with one saying: “Sometimes sharing a little bit of your own story helps.” She and another clinician discussed child rearing:

- Clinician 1: I’m an immigrant too. I can relate to them with my own experience, with my kids and then I can share a little bit of—disclose my issues.
- Clinician 2: Or empathize.
- Clinician 1: They’ll feel more comfortable and understood.
- Moderator: What kind of example specifically?
- Clinician 1: Cultural differences between parents and children. My parents don’t understand what I go through. And sometimes I say, “My son says the same thing to me. But have you understood your parents sometimes?” Or I say, “I see it because I see it in my kids, too.” And then validate their frustrations.

In this exchange, clinicians discussed patient concerns about generational conflicts involving children who were perceived as more acculturated than their parents who were presenting for treatment. The second clinician reframed the first’s self-disclosure of “issues” as empathy. Only one administrator raised concerns against therapist disclosure: “Usually clinicians are not comfortable with disclosing personal matters. I would focus more on the things perceived that we have in common.” This administrator did not specify strategies for eliciting commonalities.

Instead, all groups suggested that clinicians should explain more options to patients. All patients agreed that clinicians should enlist patients in treatment planning rather than assume automatic adherence to treatment, with one linking enlistment to clinician satisfaction: “They should take time to explain to you why you feel you need it, not just give you a prescription and demand that you take it. Every medication the doctor chose to put me on, he’s explained to me why he’s giving it to me.” Another patient valued his clinician’s explanations based on knowledge of his illness course: “When I came out [of the hospital], my doctors said, ‘You see. These are the consequences from not taking medication.’ And it got to a point where I said, ‘I’m tired of this. I understand.’ I said, ‘Okay. The medication kept me from being hospitalized.’”

Clinicians and administrators saw explanations from clinicians as opportunities for patient psychoeducation. One psychiatrist typified clinician responses: “I usually educate them. First, how important it is to have both things [medications and psychotherapy] in their treatment. Also, sometimes offering them to give it a try. ‘It’s not so bad. If you don’t like it, maybe we can try another way.’” Administrators preferred psycho-education especially for patients wanting to stop medications, with one saying: “It is important for them to be aware of all the alternatives and the risks that they are assuming when they’re withdrawing medication. At the same time, give them some tools to undo that decision. ‘Let’s make a trial of 6 months and see what happens, and then if not we will go for a second step and a third step so we can all navigate the stormy waters until we go to a safe place.’”

The limits of patient enlistment appeared with respecting patient wishes. Patients objected to being treated like others without individualized care, as in this exchange:

- Patient 1: Sometimes the doctors don’t listen to you. They should take a few minutes to listen to you before they make a decision. Sometimes they—I had doctors who were judgmental.
- Moderator: When you say “judgmental” you mean …
- Patient 1: They just treated me …
- Patient 2: Like a 100 other people.
- Patient 1: Yeah. They just put me on the first medication that came into their mind and it made me sick. I had to fight to get off the medication because it made me have seizures.

Clinicians and administrators agreed with customizing care to respect patient wishes except during acute danger or psychosis, and agreed that these are potential limits around patient-centeredness. One clinician said, “If the patient is having a psychotic episode and he’s declining all care, you just take them to the emergency room. Call 911. That’s how you solve that disagreement. If it’s chronic depression or anxiety and they’re still able to function, you start where they want to start. ‘You want to come once a month, come once a month. And then hopefully we’ll build the relationship.’”

#### Patient challenges to CCC

All groups agreed that patients bring challenges to culturally competent care. Patients and clinicians mentioned patient models of mental health care and views of mental illnesses. Clinicians and administrators mentioned patient concerns around the clinician’s culture.

The patient’s cultural model of mental health services could introduce challenges to culturally competent care. One patient typified three other patient responses: “Our country is very Spanish, so if you go to the *psiquiatra* [psychiatrist] or the *psicólogo* [psychologist], it means you are crazy. But here, a lot of people, especially white people, go to the *psiquiatra* and take pills like they get Tylenol.” Two clinicians discussed how patient treatment expectations interfere with care, with one saying: “Some patients are more likely to just want medication management. They find it much more acceptable than therapy. They think that medication makes it more of a physical illness whereas therapy means that they’re crazy.”

One patient and two clinicians mentioned patient cultural views of mental illness as a barrier to care. A patient with recurrent bipolar depression narrated her challenges with religion, as did one clinician who described similar experiences with her patient: “They were very resistant to the patient coming in and just kept dragging her all over New Jersey from one church to another church. She had a very severe bipolar disorder. She had very psychotic episodes. That was just viewed as some kind of religious punishment. She was not religious enough.” Only patients and clinicians mentioned these barriers, not administrators.

Finally, one clinician and administrator indicated that the patient’s perceptions of the clinician’s culture could introduce barriers to care. The clinician said, “Some patients reject you for who you are because we are from another culture. Eventually you work through it and you can see the result. They’re opening up and they admit, ‘Initially, I didn’t believe in you, but I think I did the right thing.’ It’s a big thing. Some patients never come back and say, ‘I want a different therapist. You won’t understand me.’” The administrator said, “We have one particular therapist from one nationality who, when given a patient with that same nationality, the patient always refuses care.” Although the administrator did not want to name the nationality in the focus group, she said that patients refused care due to concerns that medical confidentiality would not be respected in their community.

#### Clinician challenges to CCC

Two patients and three administrators—but no clinicians—identified explicit and implicit clinician biases as challenges to CCC. This exchange exemplifies explicit bias from a clinician who was blamed for exoticizing her patient’s physical traits:

- Patient 1: I had one therapist that, I’m mixed, and when I would come to see her, all she wanted to talk about was my culture, instead of dealing with my psychiatric problems, instead of dealing with the depression and what was going on at that time.
- Moderator: She wanted to talk about your culture?
- Patient 1: Yeah, and my hair and my eyes.
- Patient 2: Your therapist wanted to do that?
- Patient 1: And she would do it at every session.

Administrators also mentioned explicit biases, but in reference to “political correctness”: one administrator warned: “We may begin to behave in culturally sensitive ways that actually demeans people’s cultures. We may miss the boat either by just not paying attention to it or paying too much attention to it. If we can become culturally condescending by beginning to focus on culture—just be a good doctor with me, this other stuff is not necessary.”

Two administrators expressed concerns that clinician implicit biases could interfere with cultural competence. One administrator stated: “We all have attitudes and beliefs that we come into this practice with that we have to check at the door and be very mindful of.” Another gave an example of challenges that she faced as a clinician: “Not bringing in my own judgments, preconceived notions that I may have grown up that I have with my cliques of friends. It is constant learning about who I am and what I’m bringing to the table.” Both clinicians suggested that self-awareness could counteract biases against patients.

#### Institutional challenges to CCC

Respondents in all groups reported that time and technological pressures threatened culturally competent care. This patient exchange represented time concerns:

- Patient 1: The thing that you really want to tell them and get off your mind, by the time that half hour comes, you’re about to say it—“Ok, I’ll see you next week.”
- Patient 2: I don’t know who came up with 45 min. If we had maybe an hour, that would be great. Because it’s pretty small and they …
- Patient 1: Yeah, but then there’s too many people that they have to get to …
- Patient 2: And that’s the problem. There’s so many patients, that they have to do it and …
- Patient 1: Right.
- Patient 2: I get upset sometimes.

These patients viewed time as a scare commodity. Despite wanting time, patients understood the demands for clinicians to see other patients. Clinicians agreed with the need for more time, with one saying: “Insurance is cutting the time very short. It’s taking away what we are actually trained to practice. Patients are not getting that kind of quality care.” An exchange among three clinicians demonstrated time pressures:

- Clinician 1: Now you got to end the session because of time constraints. You can just see it in their face sometimes: “You left me hanging there.”
- Clinician 2: Using your own personal time. You don’t take breaks. And sometimes that’s not good because our health is important to give health to others.
- Clinician 3: Who’s looking out for the clinician?
- Clinician 1: You walk fast.
- Clinician 2: Walk *and* talk.
- Clinician 1: You learn through experience to wind down people. Some people are very talkative.

Clinicians regretted using personal time to accommodate patients. Based on time pressure, one administrator doubted whether culturally competent care could be achieved: “We lack the time to explore all these multiple cultural factors and variables in short sessions.”

Time efficiency led to more use of technology in appointments:

- Patient 1: Before, the therapist would look at you. But now they’re typing while you’re talking to them. You don’t feel it.
- Patient 2: The computer.
- Patient 1: I feel very uncomfortable because I feel that they’re not paying attention to me. I don’t blame them for it because there are people that say, “This is what you have to do now. Now you have to be on the computer.”

As with time, patients expressed ambivalence about their desires for attention and clinician needs for efficiency, a point emphasized by one clinician: “I find that before we jump into the computer, I explore. “What brings you here today?” I sit back, I look comfortable. I look them in the eye. I’ll explore first. It only takes like 5 or 10 min. You can build up some trust in that short period of time. And you say, “We only have a certain amount of time. I got to ask you these questions that we have listed in the computer.””

#### Hospital policies on cultural competence

The institutional standard for cultural competence is the hospital’s diversity policies. All clinicians and administrators are trained in these policies during new employee orientation and in annual refresher sessions. The policies include this definition for cultural competence:

- Diversity is much more than skin color, gender, age, religion or background. It’s internal and external. Each of us is diverse in many ways—chosen and random—and each of us brings many qualities to the world of work. No two people are identical. What makes us diverse? Many factors create diversity: personality style, thinking style, processing style, assertiveness level, religion, age, gender, race, culture, disabilities, values, energy level, habits, likes and dislikes, education and knowledge, goals and ambitions, political views, lifestyles, sexual orientation, social status, job titles, and many others. There is more to diversity than meets the eye. No two people are identical, and even if one of us is right, the other does not have to be wrong. **We must learn to accept people for who they are, not who we want them to be** [original emphasis].

The handbook also includes “steps to dealing with diversity” such as “understand and respect individual differences,” “be assertive,” “learn how others want you to treat them,” and “act as a force for change.” These steps combat “stereotypes,” defined as “when we apply our experiences with one member of a group to the entire group.” Stereotypes can “cloud the fact that *all* attributes may be found in *all* groups and individuals” [original emphases].

## Discussion

This article has analyzed cultural competence meanings and practices through focus groups at a diverse New York City hospital. Participants discussed themes around culturally competent care. Their responses were contrasted with each other and with hospital policies to explore everyday understandings against institutional standards.

Most administrators defined cultural competence through group-based, demographic traits compared to person-centered definitions. Some administrators and clinicians treated traits particular to specific groups as targets of clinical intervention. Repeated use of the word *background* in the focus groups underlies an understanding of culture in mental health as static and inherited rather than dynamic and acquired (Gaines 1994). Those who believe in this model of cultural competence may not think that clinicians can deliver culturally-competent care when patient and clinician backgrounds and identities differ. This tendency emerged among administrators who recommended patient-clinician matching by perceived cultural similarities as a model for cultural competence. Clinicians and administrators typically define patient identities by making assumptions about physical appearance based on racial or ethnic backgrounds rather than asking patients directly about their cultural identities (Aggarwal 2011). Taken to the extreme, a group-based understanding among administrators and clinicians may approach the definition for “stereotype” in hospital policies of applying experiences with an individual to an entire group. In our focus groups, participants assigned deviations from expected clinical norms for trust, eye contact, family structures, or therapeutic disclosure to racial or ethnic differences. Since all administrators and clinicians are trained in these policies, it is unclear if the group-based model for cultural competence is due to insufficient training or disagreement with policies.

In contrast, person-centered definitions focused on customizing care for each individual. This understanding is consistent with the patient-centered movement across the health disciplines since the 1980s that has prioritized respect for patient wishes in clinical interactions (Saha et al. 2008). That most patients and clinicians responded in this way suggests that these stakeholders may see cultural competence as integral to treatment through direct patient interactions compared to administrators working at systemic and organizational levels. Person-centered definitions of cultural competence as fighting stigma also indicate that suffering from mental illness may be a more salient identity than “background” identities of race, ethnicity, or language (Yanos et al. 2010). Indeed, hospital policies affirm this person-centered definition by noting that diversity is “internal and external.” The list of “factors” for diversity includes “internal” traits that cannot be determined by prioritizing “external” appearance.

Discrepancies between hospital policies and actual practice raise questions over how to align priorities around cultural competence. For example, current policies do not specify best practices for clinicians on how to conduct a comprehensive cultural assessment. Some clinicians and administrators undergoing hospital training may have espoused group-based models of cultural competence based on their personal experiences of delivering cross-cultural care rather than implementing an institutionally-endorsed alternative. One solution is to train staff in specific models of cultural assessment. For example, the fifth edition of the Diagnostic and Statistical Manual of Mental Disorders (DSM-5) published by the American Psychiatric Association includes the Cultural Formulation Interview (CFI), a 16-item questionnaire based on theories of cultural competence that have the greatest evidence base in mental health (Lewis-Fernández et al. 2014). The CFI simultaneously affirms the value of group-based and individually constructed identities in its eighth and ninth questions, preceded by a prompt:

- Sometimes, aspects of people’s background or identity can make their problem better or worse. By background or identity, I mean, for example, the communities you belong to, the languages you speak, where you or your family are from, your race or ethnic background, your gender or sexual orientation, or your faith or religion.
- 8. For you, what are the most important aspects of your background or identity?
- 9. Are there any aspects of your background or identity that make a difference to your problem? (American Psychiatric Association 2013).

The CFI recognizes that culture emerges from the communities to which one belongs, i.e., group aspects of identity. The CFI also encourages clinicians to personalize care by asking patients which aspects are most crucial while seeking care for a mental problem. This method of personalizing care so that clinicians learn about the patient’s desires and motivations beyond merely treating an illness category is one way to operationalize findings from a study in which patients belonging to American minority groups wanted providers to affirm their understandings of who the patient is in order to demonstrate clinical rapport (Mulvaney-Day et al. 2011).

All groups also mentioned that clinicians should share similarities with patients as techniques for achieving cultural competence, but patients and clinicians wanted clinicians to relate personal experiences whereas an administrator warned against professional boundary violations. Role expectations may explain differences. Administrators supervising clinicians may want to avoid breaking institutional rules whereas clinicians may be willing to experiment with different forms of interactions with patients since they ultimately are responsible for treatment. Psychologists have shown that US racial and ethnic minorities respond positively to therapist self-disclosures related to cultural identities (Burkard et al. 2006; Constantine and Kwan 2003). In this regard, our clinician sample exhibits notable differences from Willen’s (2013) study of psychiatry residents who complained about a cultural sensitivity course since instructors did not discuss how clinicians’ personal experiences with race and ethnicity affected their approaches to patient care. Indeed, many anthropological studies on cultural competence have been conducted with psychiatric trainees who have resisted instructor-led discussions of culture and difference since many trainees have their own negative experiences with cultural differences that are often unacknowledged (Willen and Carpenter-Song 2013). In contrast, our clinician sample openly discussed strategies to bridge cultural differences even when they departed from hospital policies. It is possible that differences in sample characteristics underlie our findings: we enrolled clinicians beyond only psychiatrists, independent practitioners rather than residents in training, and clinicians in a diverse community where working through cultural differences is necessary to provide services. Future work could examine perceptions of cultural competence across different types of clinicians, at various stages of practice, and in a variety of institutions such as academic, community, and for-profit hospitals.

All groups also mentioned that clinicians should explain more options to patients, but only patients and some clinicians prioritized respecting patient wishes. Patients wanted to be treated as equal partners in treatment planning whereas clinicians and administrators viewed explanations as opportunities for psychoeducation. The growth of managed care since the 1990s has led to patients seeing themselves as consumers and clinicians as consultants rather than older models of clinicians as omniscient and omnipotent (Kronenfeld 2001). Respecting patient wishes matches hospital policies to “understand and respect individual differences.” However, patients and clinicians may view medical communication differently: patients tend to evaluate clinicians based on expressions of respect and empathy whereas clinicians focus on information gathering for diagnostic and treatment planning (Aggarwal et al. 2015). Limits on the consultation model appear during acute illness when clinicians may need to treat patients against their preferences (Aggarwal 2012). One solution may be to ask patients how they wish to be treated during times of acute illness such that patient preferences and the range of clinician responses are discussed in advance of clinical emergencies.

All groups also discussed patient challenges to culturally competent care, though only administrators mentioned clinician challenges. Patient challenges included cultural understandings of mental health services whereby only “crazy” people presented clinicians and needed psychotherapy. This cultural understanding stigmatizes mental illness and may lead to treatment non-adherence (Vargas et al. 2015). Patients and clinicians also indicated that negative perceptions of each other’s cultures could impair the relationship. Clinicians detailed their attempts at introspection to counter implicit biases, using techniques similar to cultural humility where self-critique can redress clinical power imbalances (Tervalon and Murray-García 1998).

Lastly, all groups mentioned time and technological pressures as institutional challenges. The use of quality assurance and utilization management procedures throughout medicine since the 1990s has linked good clinical treatment to long-term cost controls as person-centered care has transitioned to population-based algorithms (Donald 2001). Our participants equated time constraints to decreased quality, noting that patients did not receive quality care when treated like hundreds of others. Time pressures have also been named to avoid implementing cultural competence initiatives (Aggarwal et al. 2013). Others have warned that changes in clinician knowledge, attitudes, and skills may not actually change clinician behaviors if the institutional structure of health care delivery is also not changed (Kirmayer 2012). We see time management and quality assurance as distinct domains: clinicians can individualize care in short appointments or treat people algorithmically even without time constraints. Hospital clinicians used personal time to satisfy institutional requirements, but may benefit from recognizing time management and quality assurance as distinct. Administrators could also designate one billable appointment for a cultural assessment such that patients and clinicians do not feel as if individual patient preferences are excluded, a tactic that has successfully made the business case for cultural competence in community settings (Bassiri and Soriano 2016).

Our results should be interpreted in light of several limitations. First, this study was conducted in a single hospital, potentially limiting the generalizability of findings. This is a limitation common to all single-site studies, qualitative and quantitative. The goal of qualitative research is to advance social theory by designing research rigorously that accounts for the unique perspectives of subjects and researchers through robust data analysis, not to posit cause-effect relationships as in statistically-based quantitative research (Mays and Pope 2000). We situate our work within this tradition by presenting the range of stakeholder opinions on cultural competence at one community hospital. At the same time, we have striven for representativeness by enrolling all relevant stakeholders. Intervention studies that are intended to change human behavior often narrowly select participants and apply narrowly specified strategies that can limit the representativeness of results (Glasgow et al. 2004). Our study aimed for representativeness by broadly enrolling patients, clinicians, and administrators with minimal exclusion criteria and encouraging them to brainstorm strategies for changing human behavior rather than asking them to apply strategies determined by the researchers beforehand. Future qualitative research could explore whether our findings on cultural competence converge or diverge from findings in other clinics. Second, existing relationships among clinicians and administrators could have influenced interactions. Although no single participant dominated any group, perceptions of influence may have encouraged some to participate more than others. Third, we followed methodologists in using theoretical sampling to achieve data saturation. There is no consensus among focus group methodologists on the numbers of participants needed to achieve data saturation among methodologists (Carlsen and Glenton 2011). We believe that our findings exhibit candor and exhaust the range of responses, including contradictions among participants, indicating that we have achieved data saturation.

This study offers future research, policy, and practice directions related to cultural competence. The discrepancy between employee responses and hospital policies raises several possibilities. Employees may benefit from role-specific training as administrators or clinicians that is relevant to professional responsibilities and more frequent training than an initial orientation followed by an annual session. Techniques to achieve cultural competence can also be specified within hospital policies in an ongoing fashion so that clinicians who understand policies also have actionable procedures to accomplish goals. In addition, hospital leaders could incorporate patients, clinicians, and administrators in writing policies since culturally-competent care affects multiple stakeholders. Finally, patients and clinicians could initiate discussions on how to respect patient wishes and explain treatment options in advance of treating acute illnesses. More research is needed on how clinicians should ascertain and act as consultants or as educators to patients since patients have their own models of mental health illnesses and services that do not necessarily correspond with those of providers.

## Conclusions

Our study demonstrates the value of qualitative research methods in hospitals. Hospitals remain understudied despite being preeminent domains that refract mainstream society’s core values and beliefs (van der Geest and Finkler 2004). The hospital in our study represents the challenges of a broader American society struggling to redress injustices for historically disadvantaged minorities, provide services for diverse immigrants, and balance market efficiency with consumer satisfaction. Staff meetings, employee orientations, and other hospital settings can illuminate how multiple stakeholders create and debate everyday knowledge and practice that may diverge with institutional standards. Social science research in health care settings can illuminate our understanding of how people construct institutional culture by comparing what people say with what they think they do (Lambert and McKevitt 2012). We have adopted this orientation in analyzing how patients, clinicians, and administrators construct the institutional culture of one hospital by comparing their perspectives on cultural competence (what they say) as stakeholders against actual hospital policies (what they think they do) to improve clinical practice.
